# Carbon Nanohorns Modified with Conjugated Terthienyl/Terthiophene Structures: Additives to Enhance the Performance of Dye-Sensitized Solar Cells

**DOI:** 10.3390/nano7100294

**Published:** 2017-09-25

**Authors:** Daniel Iglesias, Pedro Atienzar, Ester Vázquez, María Antonia Herrero, Hermenegildo García

**Affiliations:** 1 Departamento de Química Orgánica, Facultad de Ciencias y Tecnologías Químicas, Universidad de Castilla-La Mancha, 13071 Ciudad Real, Spain; d.iglesias.asperilla@gmail.com (D.I.); ester.vazquez@uclm.es (E.V.); mariaantonia.herrero@uclm.es (M.A.H.); 2 Instituto Regional de Investigación Científica Aplicada (IRICA), Universidad de Castilla-La Mancha, 13071 Ciudad Real, Spain; 3 Instituto Universitario de Tecnología Química CSIC-UPV, Universitat Politécnica de Valencia, Av. de los Naranjos s/n, 46022 Valencia, Spain; pedatcor@itq.upv.es

**Keywords:** carbon nanohorns, dye-sensitized solar cells, terthienyl dyes, terthienylphenylene vinylene dyes

## Abstract

A series of carbon nanohorns (CNHs) constituted by the aggregation of about 2000 individual conical graphene tubes (diameters from 2 nm to 5 nm and a length of 40–50 nm) that have been modified with dyes of two, three, or four terthienyl groups has been prepared by adsorbing the corresponding dye on the CNH. Persistent inks in *o*-dichlorobenzene (*o*-DCB) of these dye-CNH conjugates were obtained by laser irradiation of *o*-DCB suspensions of the dye-CNH solids. These inks were used in combination or not with N719 dye for the preparation of dye-sensitized solar cells (DSSC) of TiO_2_. It was measured that the terthienyl dye with the largest conjugation deposited on the CNH additively increased the performance of an analogous TiO_2_ cell from an efficiency of 4.07% to 6.24%. This result shows the potential of dye-modified CNHs as additives in the construction of more efficient DSSCs.

## 1. Introduction

There is a continuous interest in improving the efficiency of dye-sensitized solar cells (DSSCs), to reach values that can make this type of device closer to commercial application [1,2,3,4]. Among the strategies that have been reported to achieve better performance, one that is attracting current attention is the use as additives of carbon allotropes in combination with TiO_2_ [5]. Carbon allotropes such as carbon nanotubes (CNTs) [6], carbon nanohorns (CNHs) [7,8], and carbon-based materials can increase the electron mobility in the photoactive layers, and also can assist charge separation. While there are abundant precedents in the literature reporting the influence on DSSCs of CNTs and graphene, application of CNHs as a booster of the efficiency in DSSC is considerably scarcer [5,8,9,10,11]. Compared to CNTs, CNHs offer several advantages, including metal-free synthesis, higher sample purity, and larger availability due to their easier scalability [12,13]. Therefore, it would be of interest to evaluate the potential of CNHs as additives in the preparation of DSSCs to contrast their behavior with that of CNTs. The morphology of a primary CNH particle is a single conical graphene tube from 2 nm to 5 nm in diameters and a length of 40–50 nm, with a hemispherical-closed tip (in contrast to the cylindrical structure to single-walled CNTs that exhibit much longer length in the micrometer scale). Around 2000 individual CNH cones aggregate together, forming a spherical dahlia flower-like structure with a rather narrow diameter distribution of the ensemble of about 80–100 nm. Note that CNTs form bundles of random shape and much higher length as compared to the better structuring and arrangement of CNHs. One of the main problems associated with the use of CNHs is the low dispersability in liquid phase of the structured aggregates as a consequence of their morphology and size [14].

In a recent study reported by some of us [8], it has been shown that CNHs as an additive increase the efficiency of DSSC devices to a greater extent than when using CNTs and graphene as additives. Based on this precedent and considering that there are other ways of modifying CNHs, in the present manuscript we went a step further by exploring the performance of DSSCs in which CNHs have been modified by adsorbing terthienyl dyes with the purpose of combining in a single additive the previously observed advantages of electrical conductivity introduced by CNHs with the light harvesting properties of the dyes. These studies will expand the scarce application of CNHs in photovoltaic devices and will serve to illustrate the advantages of CNHs with respect to other carbon allotropes. Two types of CNHs, pristine or oxidized (to increase the density of carboxylic groups) CNHs, were used to adsorb terthienyl molecules. It is known that carboxylic groups establish a strong interaction with titania surface, and this interaction is responsible for the higher efficiency of metal polypyridyl complexes with carboxylic substituents as photosensitizers in DSSCs [1]. For this reason, it could also be possible that chemically modified CNHs with carboxylic acid groups would strongly bind to TiO_2_ favoring electron transfer processes. On the other hand, polythienyl dyes with arylenevinylene moieties have also been widely used as light harvesters and electron donors in photovoltaic devices [15,16]. Therefore, it is of interest to determine if it is possible to combine in a hybrid material comprising CNHs and terthienyl dyes the light harvesting and conductivity properties required to develop DSSCs with enhanced efficiency. The selection of these dyes is based on their good processability and their high extinction coefficients in the visible region.

## 2. Results and Discussion

**Figure 1:**
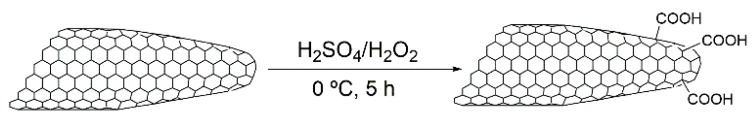
Chemical oxidation of carbon nanohorn (CNH) samples generating carboxylic acid groups.

**Figure 2:**
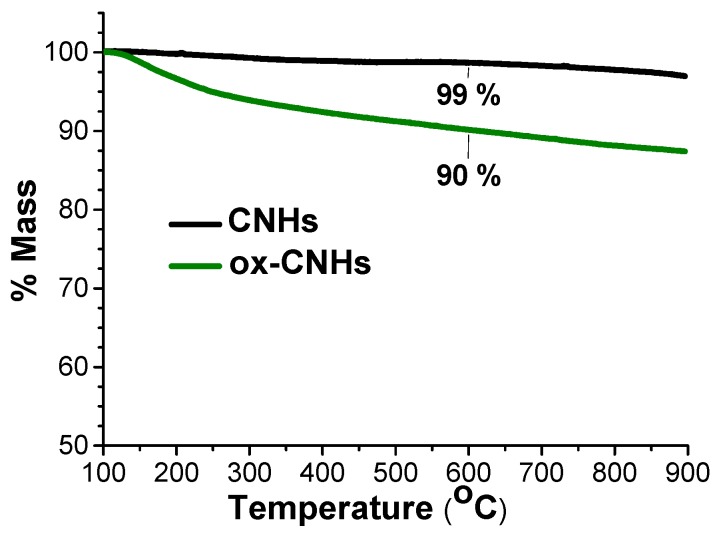
Thermograms of CNHs before (black) and after oxidation treatment (green). The difference in the weight loss confirms the functionalization.

Commercial CNHs samples were oxidized by sulfuric acid and hydrogen peroxide (see Figure 1). Oxidation in strong acid media is well established in CNTs and active carbons as a way to introduce carboxylic acid groups on graphitic walls [17,18]. Thermogravimetric analysis before and after the oxidative treatment are shown in Figure 2. The success of the oxidative protocol is confirmed by the increase of the weight loss at high temperatures. These analyses allow the estimation of the degree of functionalization that in this case is 0.2 mmol of –COOH groups per gram of material.

**Figure 3:**
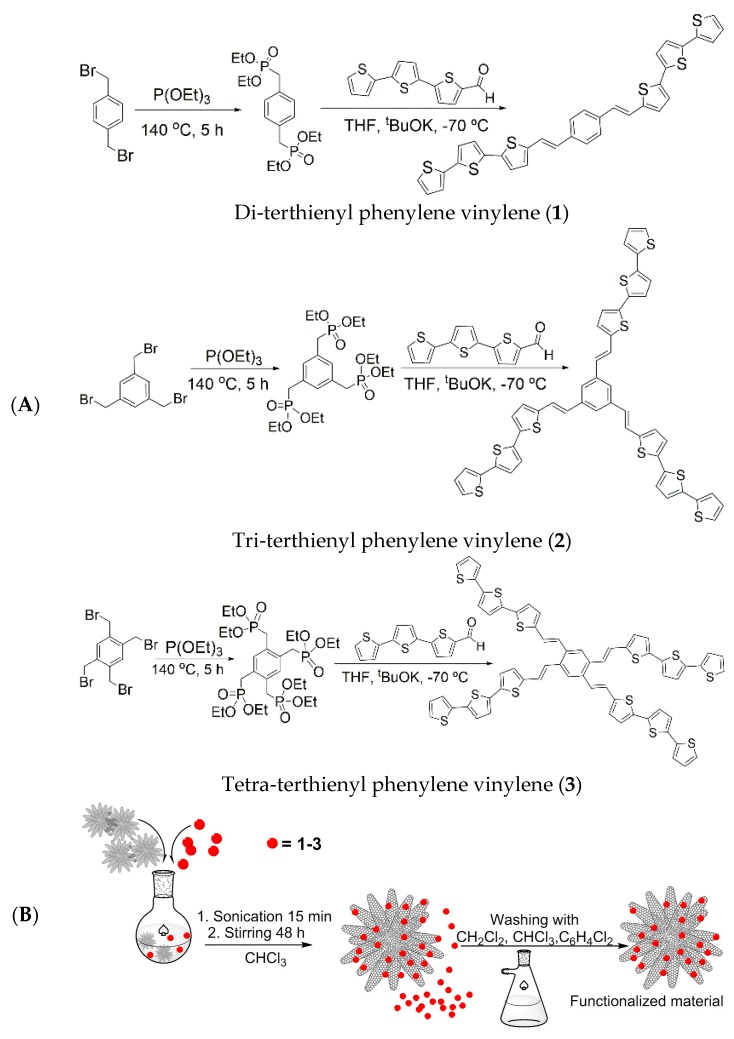
Synthetic route of CNH samples having adsorbed terthienyl dyes: (**A**) Synthesis of dyes (**1**)–(**3**) that will form non-covalent aggregates with CNHs; (**B**) pictorial illustration of the process used to attach the dyes to CNHs.

The terthienyl and phenylenevinylene dyes were obtained by condensation of *p*-(diethoxy phosphonylmethyl) benzene (for dye **1**), 1,3,5-tris(diethoxyphosphonylmethyl) benzene (for dye **2**), and 1,2,4,5-tetrakis(diethoxyphosphonylmethyl) benzene (for dye **3**) with 2-carbaldehyde-2,5-terthienyl under basic conditions. Figure 3 illustrates the synthetic route followed to obtain the corresponding dyes. A full description and characterization of the dyes was reported separately [19]. The low solubility of these kind of dyes is a drawback that makes difficult their proper characterization by ^1^H-NMR or ^13^C-NMR spectroscopy, but in owner case a complete NMR study was published. Such insoluble compounds are typically identified by Fourier transform-infrared (FT-IR) spectroscopy and matrix-assisted laser desorption/ionization-time of flight (MALDI-TOF) mass spectrometry for which this set of compounds showed also physical and spectroscopic properties in agreement with those corresponding to their structure [20,21,22].

**Figure 4:**
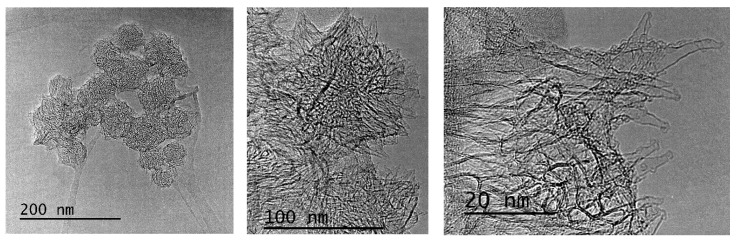
Transmission electron microscopy (TEM) images of dye-CNH **3** where the presence of individual dahlia-shaped particles can be observed.

The morphology of dye-CNH conjugates was studied by transmission electron microscopy (TEM) images. Figure 4 show representative images of the dye-CNH **3** sample used in the present study in where the characteristic dahlia-like morphology of CNHs can be observed.

**Figure 5:**
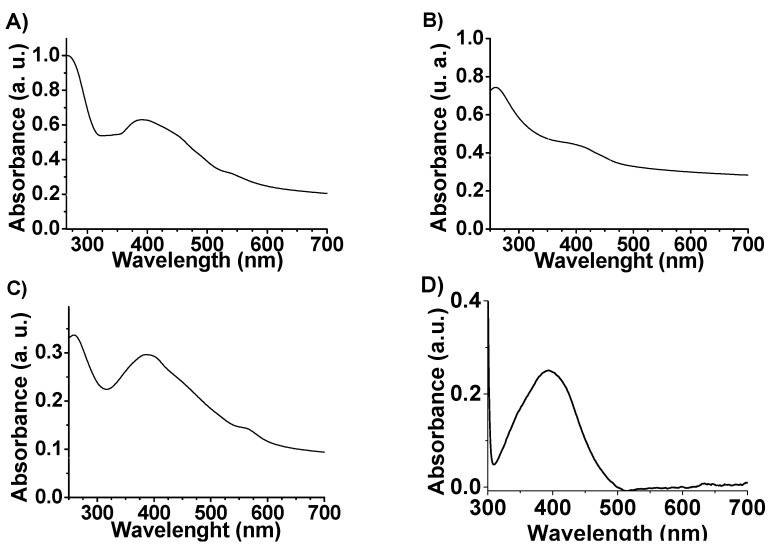

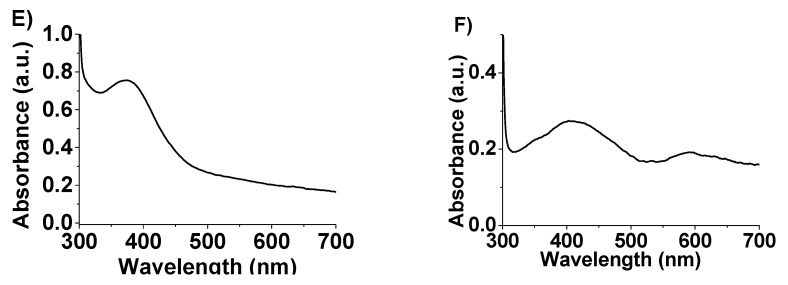
UV-Vis absorbance spectra of dye-CNHs **1**–**6** (**A**–**F**) in 1,2-dichlorobenzene.

For the purpose of dye sensitization, one of the most relevant properties is the UV-Vis spectra of the dyes. Figure 5 shows the optical absorbance of the dyes adsorbed on CNHs that are characterized by absorption maxima between 390 nm to 600 nm, where the maximum absorbance is red shifted depending of the number of thiophene units. Also, the two characteristic bands of the non-functionalized dyes are combined in a single broad band, probably because of the electronic interaction with the graphitic walls of the CNHs. As expected, the molar absorptivity and the position of the λ_max_ wavelength not only depends on the numbers of thiophene units, but also on the substitution pattern. In a previously mentioned report, the correspondence between these two parameters have been studied by UV-Vis spectroscopy and computational modelling [19].

**Figure 6:**
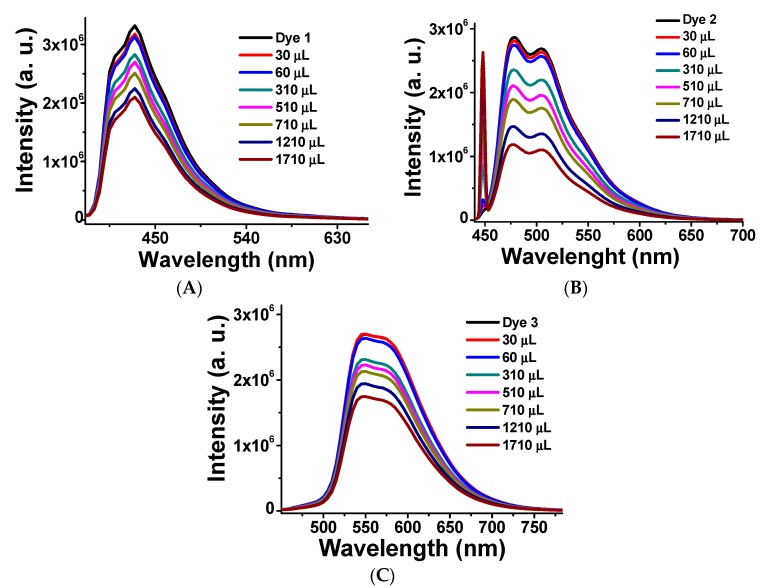
Fluorescence spectra of dyes **1** (**A**) (λ_ex_ = 363 nm); **2** (**B**) (λ_ex_ = 409 nm), and **3** (**C**) (λ_ex_ = 442 nm) upon addition of increasing amounts of CNHs (0.01 mg/mL). The solvent of these measurements was DMF and the concentration of the organic compound constant during the experiment.

Association of flat, aromatic organic molecules with graphene walls of CNHs and graphene materials as a consequence of the strong π–π interactions is a well-known fact [23]. Pyrene, porphyrins and phthalocyanines are among the preferred dyes that have been reported to form this type of non-covalent association complexes with graphene walls [23,24,25,26]. In addition, there are precedents in the literature describing the interaction of phenylenevinylene dyes with CNTs [27,28]. In the present case, association of terthienylphenylene vinylene dyes with CNHs with or without carboxylic acid groups was observed by small shifts in the position of λ_max_ upon addition of increasing amounts of CNHs, as well as by fluorescence quenching of the characteristic emission of the dyes upon addition of increasing concentration of CNHs (Figure 6). It should be noted that at the concentrations used in the photoluminescence quenching experiments the internal filter effect of CNHs should be negligible. This quenching emission demonstrates the electronic interaction between the CNHs and the different dyes. Similar behavior has been observed and discussed in other related systems [29].

**Figure 7:**
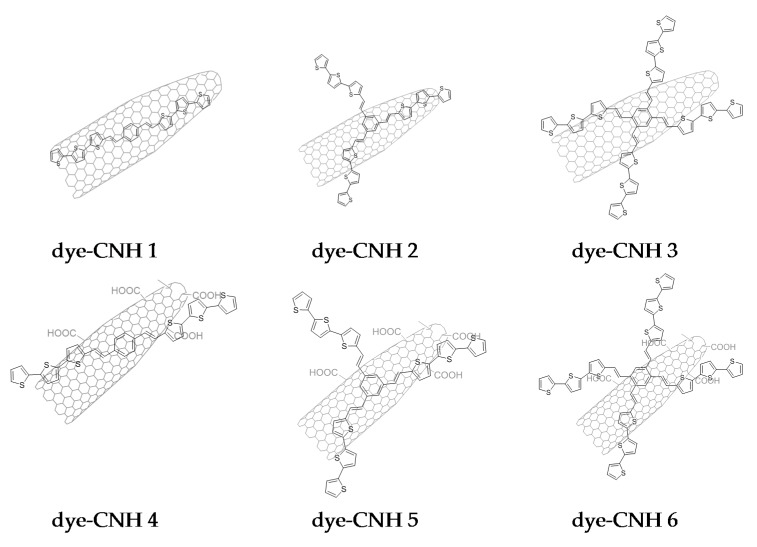
Codes and components of the functionalized CNHs used in the present work.

This noncovalent interaction between terthienyl dyes and CNHs allows the preparation of materials containing both components. Figure 7 contains the list of materials that have been tested as additives in the preparation of titania DSSC devices.

**Figure 8:**
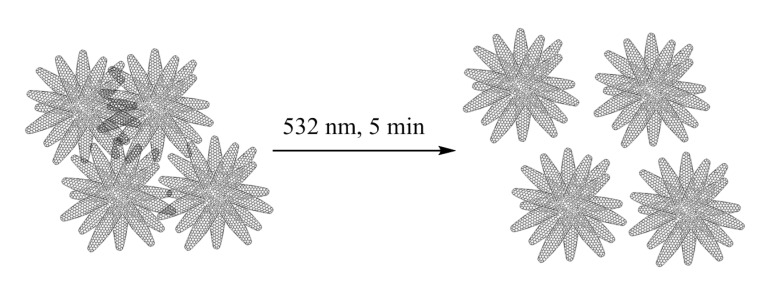
Effect of laser irradiation in CNH aggregates.

**Figure 9:**
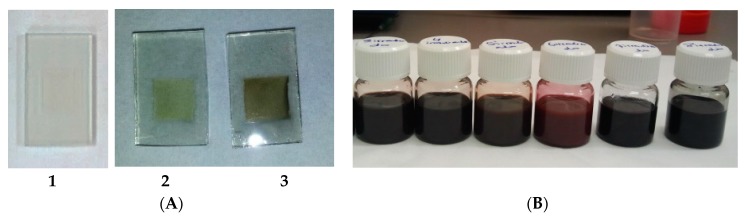
(**A**) **1**. TiO_2_ Film; **2**. TiO_2_ film sensitized using dye-CNH solution not irradiated and **3**. TiO_2_ sensitized using laser irradiated dye-CNH solution and (**B**) Photograph of *o*-DCB suspension of dye-CNHs **1**–**6** (from left to right).

A remarkable observation was that aggregates containing terthienyl dyes and CNHs cannot be dispersed easily in liquid media. We and others have observed previously that submitting carbonaceous materials, including CNTs and graphene, to laser pulses in the presence of a solvent increases dramatically the amount of material dispersed and the persistence of the resulting suspensions [30]. With this background, dye-CNHs **1**–**6** having adsorbed dyes on CNHs suspended in *o*-DCB were submitted to 532 nm laser pulses (7 ns FWHP, 50 mJ·pulse^−1^) in the absence of oxygen for 5 min. In agreement with our expectations, after this time of irradiation, persistent inks of dye-CNH conjugates were obtained. These inks were subsequently used, after removal of any remaining solid residue, for infiltration/impregnation of TiO_2_ films during the fabrication of dye-CNH modified DSSCs. Dynamic laser scattering and measurements of zeta potential of the samples **1**–**6** suspended in *o*-DCB show that the laser pulses produced a decrease of the Zeta potential from −12.1 mV to −17.8 mV, as well as a reduction of the dynamic dimensions of the suspended particles from 235 nm to 184 nm on average, both experimental measurement indicating that the suspension has increased in stability (more negative Z-potential) and is constituted by somewhat smaller particles. These two facts are beneficial from the point of view of the persistency of the suspension. We suggest that the mechanism through which the laser pulses produces these effects is by thermalization of the energy of the photons on the surface of dahlia-shape aggregates as well as the effect of mechanical shock waves produced by the laser pulse. The possibility that electrostatic repulsion due to the occurrence of photoinduced electron transfer as consequence of the photoexcitation also plays a role on the increase in the stability of the dye-CNH conjugates suspension cannot be excluded. Figure 8 describes our proposal to rationalize the effect of laser excitation on the dye-CNH conjugates. In addition, we note that an increase of the degree of TiO_2_ film sensitization using inks of dye-CNHs prepared by laser irradiation was observed. Figure 9A shows a comparative photograph of TiO_2_ films, without dye-CNH sensitization (**1**) and films sensitized by using dye-CNH inks prepared without (**2**) or with laser stabilization (**3**). Figure 9B also shows a photograph of the dye-CNHs **1**–**6** inks used in the present study.

**Figure 10:**
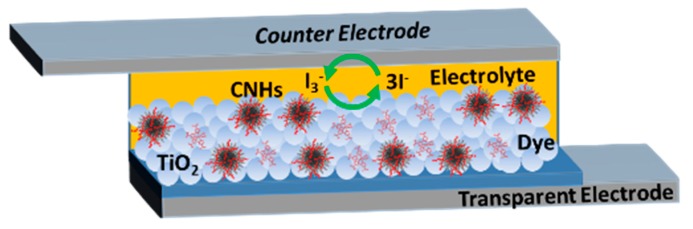
Configuration of a dye-sensitized solar cell (DSSC) using CNHs as additive.

The inks of dye-CNHs **1**–**6** after exposure to 532 nm laser pulses and removal of the remaining residues were persistent for periods longer than weeks without observation of sedimentation of any carbonaceous residue. These persistent *o*-DCB inks were used for impregnation of TiO_2_ thin films prepared by razor blade spreading on a clean conductive FTO electrode (Fluorine doped Tin Oxide glass substrate) of a commercial TiO_2_ paste (Dyesol). The resulting porous TiO_2_ film was calcined at 450 °C under air to remove the organic material and sinter the TiO_2_ nanoparticles. Impregnation of functionalized dye-CNH conjugates **1**–**6** was carried out overnight and the films were rinsed and used for the fabrication of a series of DSSC devices. A solution of I^−^/I_3_^−^ in 3-methoxypropionitrile was used as electrolyte and platinized FTO as the counter electrode. For the sake of comparison, we also tested an analogous DSSC device prepared under identical conditions, but in the absence of CNH additives. Figure 10 shows the configuration of the DSSCs employed in the present study. The devices (0.5 × 0.5 cm^2^) were illuminated with simulated sunlight through an AM1.5 filter at 100 mW/cm^2^ and connected to a potentiostat that allowed us to determine the open-circuit voltage (Voc), current density at short-circuit (Jsc), and fill factor (ff). The data obtained are summarized in Table 1.

**Figure 11:**
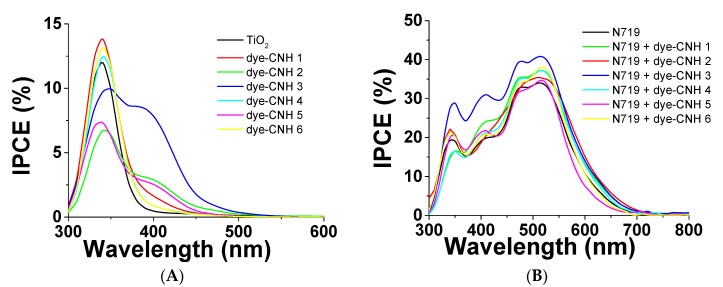
Photoresponse spectra plotted as incident photon-to-current efficiency (IPCE). (**A**) Without N719 dye sensitizer and after sensitization with N719 dye (**B**).

The main characteristic of these cells is the low efficiency as a consequence of the absence of efficient dye sensitization. It should be commented, however, that one of the cells containing CNHs was clearly more efficient than the reference TiO_2_ cell. The photoresponse spectra of the cells were obtained using a xenon lamp as the light source and a monochromator to select the wavelength. The corresponding incident photon-to-current efficiency (IPCE) spectra are shown in Figure 11. As it can be seen in this Figure, three of the dye-CNHs samples (**1**, **4**, and **6**) have a photoresponse spectrum almost coincidental with that of the reference cell prepared with plain TiO_2_. This is in agreement with the low dye content of these three samples. On the other hand, dye-CNHs **2**, **3**, and **5** exhibit in their photoresponse spectrum the influence of the presence of terthienyl armed dyes and the visible light photoresponse is in accordance with the optical spectrum of the dyes.

**Figure 12:**
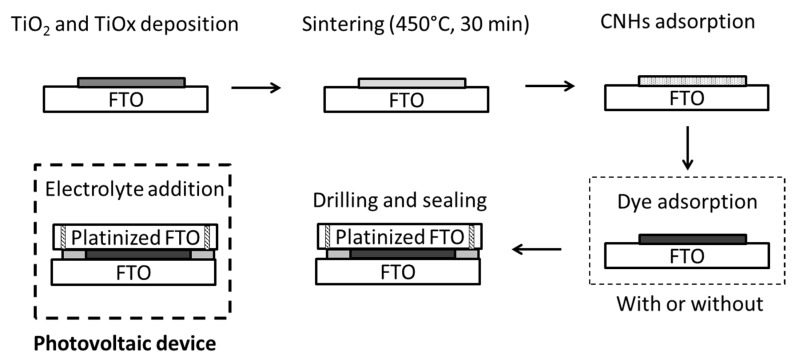
Preparation of DSSCs having dye-CNHs as additives.

Due to the low efficiency of the DSSCs built with TiO_2_ and CNH samples **1**–**6**, a second set of samples was prepared in which, after impregnation with the *o*-DCB suspensions of CNH samples **1**–**6**, the photoactive TiO_2_ layer was additionally submitted to impregnation with N719 in acetonitrile/tert-butanol (see Figure 12). As expected, this second set of DSSCs exhibited considerably higher efficiency than in the case of the reference, which was 4.07%, a result in the range of the expected value for this type of device. It should be noticed that in the cells under study, the film thickness was not optimized and the thin scattering layer on top of FTO is absent. The performance data of the set of samples prepared is also compiled in Table 1. The main feature of the data presented in this table is that the efficiency of the reference DSSC device based on N719 excitation of TiO_2_ has been improved in three cases using CNH-containing additives. Specifically, two of the best performing samples were those containing the terthienyl dye, these samples having strong absorption due to their extended conjugation. Figure S1 in the supporting information shows the photo response of IPCE and the accumulated current for dye-CNH **3** in the absence and in the presence of N719 co-sensitization. Another observation is that the experimental data did not reveal in the present case advantages derived from the oxidation of the CNHs to introduce carboxylic acid groups. In a prior study about the influence of CNHs without dyes on the performance of DSSCs, a positive influence on the IPCE of the presence of carboxylic acid groups introduced by CNH oxidation was observed [8]. Although electron impedance spectroscopy could provide further information, it seems that the presence of adsorbed dyes makes the influence of carboxylic acid groups negligible, probably due to the fact that intact graphene walls of CNHs interact better with the dye than defective oxidized CNHs. In addition, for all terthienylphenylene vinylene dyes the LUMO energy level [31] was expected to be between the conduction band of the TiO_2_ (−4.21 eV) and the LUMO level of the N719 dye (−3.01 eV [32]). Therefore, this can explain a better alignment of the energy potentials, enabling electron transfer from N719 dye to TiO_2_. Figure S2 in the supporting information provides a scheme showing the energy of the different levels involved in the in the photovoltaic response of the systems comprising terthienyl dyes-CNH and N719. With respect to the structure of terthienyl dyes, it becomes apparent that *para* substitution of the arylenevinylene arms is more favorable with respect to the meta-substitution, probably due to the extended delocalization of the π-system for the *para* isomer. This would be the most likely reason of the poor performance of dye-CNH **2** compared to the other dyes. This assumption would require, however, characterization of electron mobility and lifetime of charge separated states for a more detailed insight into the reasons of the different performance of the terthienyl dyes. Also, no correlation was observed between the IPCE and the degree of dye functionalization determined by TGA measurements that was 732, 335, and 480 μmol dye/g CNHs for samples **1**‒**3**, respectively [19].

## 3. Materials and Methods

### 3.1. Electron Microscopy Characterization

TEM images were recorded in a Philips CM300 FEG system with an operating voltage of 100 kV.

### 3.2. Preparation of the CNHs Samples

Pristine CNHs were produced by Carbonium s.r.l., Padova (Padua, Italy) by direct graphite evaporation in Ar flow, according to a patented method [33] and used without purification.

The functionalized CNHs were synthesized using the synthetic route depicted in Figure 3B. Pristine CNHs were mixed with the thiophene derivatives, sonicated and stirred. Then, the reaction mixture was filtered and washed with different solvents. The obtained compounds were fully characterized by using a range of techniques including thermogravimetric analysis (TGA), UV-Vis spectroscopy, Raman spectroscopy, fluorescence spectroscopy, and transmission electron microscopy (TEM) [19].

### 3.3. Preparation of the CNHs Inks

**Scheme 1:**
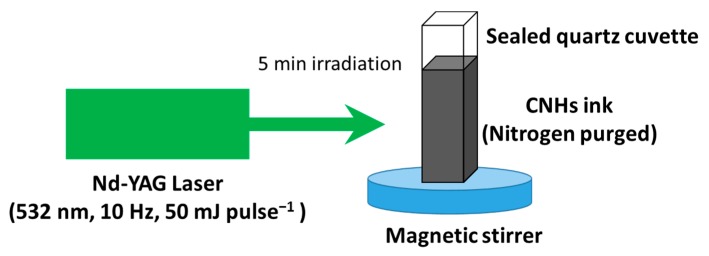
The setup for CNHs inks preparation.

CNHs powder samples were stirred magnetically and ultrasonicated for 5 min in *o*-dichlorobenzene (5 mg/mL) in a quartz cuvette and exposed to the pulses from the second harmonic of a Nd:YAG laser (50 mJ·pulse^−1^) for 5 min (see Scheme 1).

### 3.4. Preparation of the Photovoltaic Devices

A commercial titania paste (Dyesol, 18NR-T, Roma, Italy) was spread onto freshly cleaned transparent conductive glass (FTO) and the layer was sintered in an oven at 450 °C maintained for 30 min. After cooling to 50 °C, the films were immersed in 5 mL of dye-CNHs inks (5 mg/mL) overnight. For ruthenium dye sensitization, the film was also immersed in 5 mL of a 0.5 mM solution of commercial N719 dye (Solaronix, Aubonne, Switzerland) in 3:1 acetonitrile/tert-butanol overnight. The electrode was assembled with the platinized transparent conducting glass counter-electrode (FTO) using a double-sided adhesive polymer film (Surlyn, DuPont, Le Grand Saconnex Geneva, Switzerland) that acts as the separator and sealing element. The two electrodes were held together by hot melting the Surlyn seal at 100 °C while applying pressure. The electrolyte (0.5 M lithium iodide, 0.05 M iodine, and 0.4 M 4-tert-butylpyridine in 3-methoxypropionitrile) was introduced into the cell through two holes that were drilled in the counter electrode.

### 3.5. Photovoltaic Response Measurements

To determine the J_SC_-V_OC_ plots, the cell was connected to a SourceMeter (Keithley 2601, Instrumentos de Medida, S.L., Madrid, Spain). The voltage scan was controlled using ReRa Tracer software. The data were automatically transferred to a PC that controlled the experiment and at the same time provided data storage capability to the system. The solar simulator (Sun 2000, ABET Technologies, Milford, CT, USA) was equipped with an AM 1.5G filter and the nominal power for the measurements was 100 mW/cm^2^. The same cells were used to record the IPCE spectra. In IPCE measurements the sample was excited with a 150 W xenon lamp through a Czerny-Turner monochromator. The current output at short circuit was measured by a potentiostat (AMEL, Milano, Italy) which transferred the data through the A/D converter to the PC controlling the monochromator apparatus. IPCE curves were calculated using a Newport (818-UV-L) calibrated photodiode (Lasing, S.A., Madrid, Spain).

## 4. Conclusions

The present data reinforce previously reported measurements showing that carbon nanohorns as additives can increase the performance of TiO_2_ DSSCs. It has been shown that this effect can be further enhanced by the use of dye-CNH conjugates. The general drawback of coarse dispersions of CNHs resulting in CNH particle sedimentation has been satisfactorily solved by a laser irradiation pretreatment that leads to the formation of persistent dye-CNH conjugate inks as consequence of disaggregation of the CNHs. No additional benefits derived from CNHs functionalization by carboxylic acids were observed in the present case, the most efficient terthienyl dyes being those with extended conjugation and *para* substitution. Overall, the present study contributes to a rather unexplored area, showing the potential of CNH materials as additives in DSSCs.

## Figures and Tables

**Table 1:** Performance data for the photovoltaic devices prepared with dye-CNHs samples as additives in the absence or presence of N719 dye.

Sample	Voc (Volt.)		Jsc (mA/cm^2^)		Ff		Ef (%)	
	Without	With	Without	With	Without	With	Without	With
Reference	0.314	0.761	0.208	7.20	0.444	0.74	0.03	4.07
dye-CNH **1**	0.286	0.795	0.274	7.74	0.384	0.67	0.03	4.14
dye-CNH **2**	0.220	0.635	0.157	8.28	0.323	0.67	0.02	4.50
dye-CNH **3**	0.486	0.808	0.533	10.37	0.476	0.75	0.12	6.24
dye-CNH **4**	0.431	0.785	0.205	8.20	0.527	0.72	0.05	4.63
dye-CNH **5**	0.472	0.797	0.226	7.48	0.624	0.72	0.07	4.29
dye-CNH **6**	0.431	0.796	0.204	7.77	0.532	0.72	0.05	4.47

## Supplementary Materials

The photoresponse spectra of dye-CNH3 in the absence and in the presence of N719 dye (Figure S1) and energy diagram of the levels involved in the photosensitization (Figure S2) are available online at http://www.mdpi.com/2079-4991/7/10/294/s1.
