# Supplementary material for: Carbon Nanohorns Modified with Conjugated Terthienyl/Terthiophene Structures: Additives to Enhance the Performance of Dye-Sensitized Solar Cells
Source: Nanomaterials (Basel). 2017 Sep 25;7(10):294. doi: 10.3390/nano7100294 (PMC5666459; doi:10.3390/nano7100294)
Supplement: Supplementary file 1 [file nanomaterials-07-00294-s001.pdf]

Supporting information

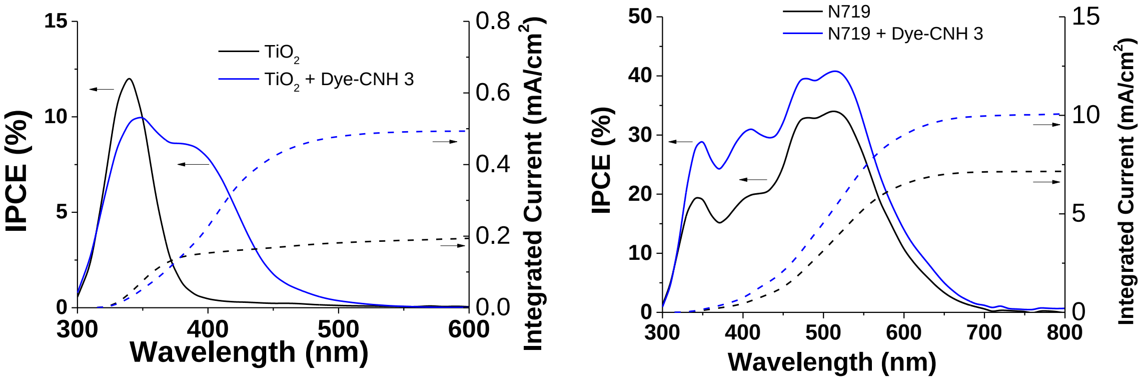

A B

Figure S1. Photoresponse spectra plotted as IPCE (solid lines, left Y) using the dye-CNH 3. (A) Without N719 dye sensitizer and after sensitization with N719 dye (B), including the current densities calculated by integrating the IPCE with the AM1.5G solar spectrum (dashed lines, right Y).

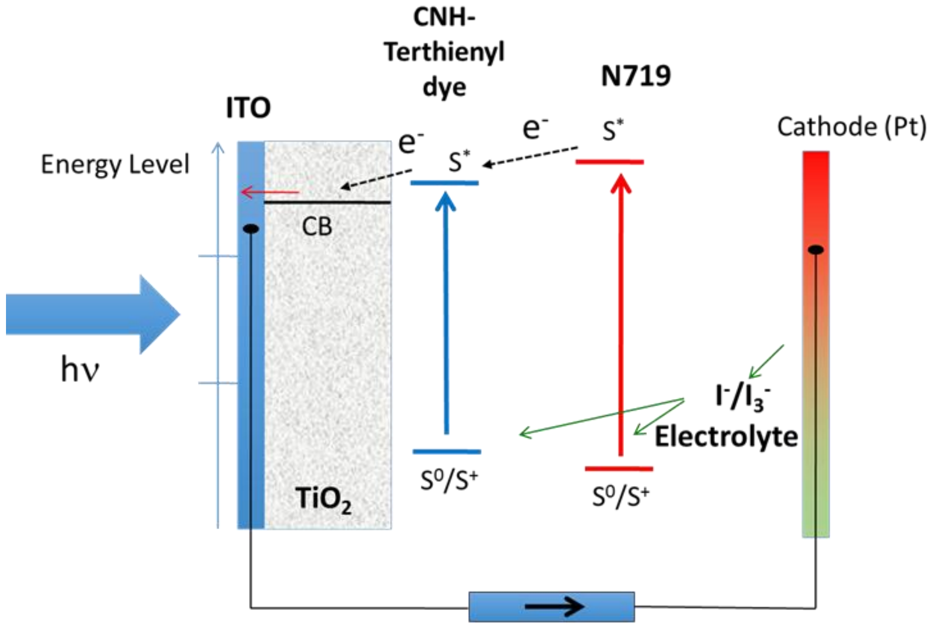

Figure S2. Diagram of energy levels involved in a dye-sensitized solar cell containing terthienyl dye-CNHs and N719 dye.
